# Prognostic Differences of Adjuvant Radiotherapy in Breast Cancer Cohorts Based on *PRLR* Genotypes, Expression, and Transcriptional Network Regulation

**DOI:** 10.3390/cancers17142378

**Published:** 2025-07-17

**Authors:** Floor Munnik, Kelin Gonçalves de Oliveira, Christopher Godina, Karolin Isaksson, Helena Jernström

**Affiliations:** 1 Department of Clinical Sciences Lund, Oncology, Lund University Cancer Center/Kamprad, Skåne University Hospital, Lund University, Barngatan 4, 222 42 Lund, Sweden; floor.munnik99@gmail.com (F.M.); kelin.goncalves_de_oliveira@med.lu.se (K.G.d.O.); christopher.godina@med.lu.se (C.G.); 2 Division of Surgery, Department of Clinical Sciences in Lund, Lund University, 221 85 Lund, Sweden; karolin.isaksson@med.lu.se; 3 Department of Surgery, Skåne University Hospital, 291 85 Kristianstad, Sweden

**Keywords:** breast cancer, PRLR, polymorphisms, biomarker, radiotherapy, prognosis

## Abstract

**Background:** Prolactin receptor (PRLR) signaling affects breastfeeding and potentially breast cancer treatment response. **Methods:** The prognostic impact of 20 *PRLR* single nucleotide polymorphisms (SNPs) in relation to adjuvant treatment groups in patients with primary breast cancer (*n* = 1701, 2002–2016, Sweden) was evaluated. Genomic DNA was genotyped on Illumina OncoArray, and survival analyses with up to 15-year follow-up were performed. Interaction models, adjusted for potential confounders, were created with different adjuvant treatment modalities: chemotherapy, radiotherapy, tamoxifen, and aromatase inhibitors. **Results:** Five SNPs (rs7734558, rs6860397, rs2962101, rs7732013, and rs4703503) showed interactions with radiotherapy and were utilized to create seven combined genotypes: six unique and one ‘rare’. Patients carrying combined genotype AG/GG/TT/CC/TC or ‘rare’ combinations derived greater benefits from radiotherapy than other patient groups (both HR_adj_ ≤ 0.29, Bonferroni-adjusted *P*_int_ ≤ 0.039). Expression Quantitative Trait Loci (eQTL) analysis revealed that three *PRLR* SNPs were associated with decreased *PRLR* expression. To explore potential SNP-associated effects, gene expression and transcriptional networks were analyzed in the METABRIC cohort and indicated that *PRLR*-low tumors were associated with reduced DNA repair signaling and enhanced anti-tumoral immunity. **Conclusions:** PRLR merits further evaluation as a putative pharmacogenomic biomarker in relation to radiotherapy for breast cancer patients.

## Simple Summary

Prolactin receptor (PRLR) signaling affects breastfeeding and potentially breast cancer treatment response. Thus, 20 normal variants in the *PRLR* gene were investigated in 1701 breast cancer patients with different treatments. Patients were followed for up to 15 years. Non-radiotherapy-treated patients with variant *PRLR* genotypes had poorer prognosis than patients with the common genotype. In contrast, radiotherapy-treated patients with variant *PRLR* genotypes experienced a better-than-expected prognosis compared to patients with the common genotypes. Utilizing a different cohort, we found that *PRLR* expression in the breast tumor alters how well the cancer can repair its DNA after radiotherapy and also plays a role in activating the immune system against the tumor. This could lead to the better effect of radiotherapy treatment against breast cancer if the tumor has low *PRLR* expression or if the patient carries certain *PRLR* normal gene variants. Our findings merit further investigation.

## 1. Introduction

Breast cancer is diagnosed in more than two million women worldwide annually [1]. Despite improvements in breast cancer treatment, 13–34% of patients experience recurrences up to 20 years after initial diagnosis [2]. Conversely, patient overtreatment has become increasingly relevant due to side-effects and the financial burden on society, thereby underlining the need for biomarkers of treatment efficacy to better guide therapeutic plans [3,4]. In the past decades, breast cancer treatment decisions have been steered by factors including age, tumor size, receptor status, tumor grade, and the involvement of axillary lymph nodes [5]. Considering the recurrence rate, a better understanding of additional factors, e.g., lifestyle, body shape, single nucleotide polymorphisms (SNPs), and other genetic variations, is essential. Besides age, host factors are rarely employed to guide the selection of breast cancer treatment despite their known influence on therapeutic effects [6,7,8].

Currently, most patients with early-stage invasive breast cancer undergo surgery and receive multiple types of adjuvant therapy. Systemic adjuvant treatment can be either pre-operative, designed to shrink large tumors before surgery, or post-operative, to lower recurrence risk. According to Swedish guidelines, post-operative radiotherapy is primarily recommended for patients who have undergone breast-conserving surgery, have axillary lymph node metastasis, or have extensive tumors [5]. Radiotherapy works by inducing DNA damage to the targeted tissue and stimulating an immune response [9,10]. Available systemic treatments include chemotherapy and HER2-targeted therapies, and for patients with ER-positive (ER+) tumors, endocrine treatment (e.g., tamoxifen and aromatase inhibitors) is commonly recommended [5].

Genetic determinants of breast cancer treatment response are promising tools in the optimization of patient outcomes across diverse therapeutic regimens. In 2007, a 60–80% increase in breast cancer-related mortality rate was observed in first-degree relatives of patients with aggressive breast cancer, demonstrating a significant association between prognosis and heritability [7]. Subsequently, several clinically relevant SNPs affecting genes implicated in drug transport, metabolism, and DNA repair were identified [8]. Despite their potential prognostic application, clinical translation of these findings remains limited, and further assessment of the impact of genetic factors on patient outcomes is warranted.

The prolactin (PRL) receptor (PRLR), known to play a role in metastasis, is another contributing factor to patient prognosis in breast cancer [11,12]. The *PRLR* gene, located on chromosome 5, can produce several transcripts through alternative gene splicing [13,14,15]. The PRLR protein and its ligand, PRL, are essential for milk production and breastfeeding. They are also implicated in a wide variety of biological processes, such as immune response and DNA repair [16,17]. PRL levels first rise during pregnancy and breastfeeding, subsequently decreasing below pre-pregnancy levels [18]. Aside from its biological relevance, emerging evidence suggests a possible association between PRLR-linked reproductive factors, such as breastfeeding duration, and breast cancer risk and prognosis [19,20,21,22,23]. Regardless, it is still uncertain whether this is due to breastfeeding-induced biological changes or underlying genetic variation.

One way that PRLR can associate with the modulation of therapeutic response is through the activation of intracellular JAK/STAT signaling after PRL binds to PRLR dimers, leading to the regulation of the target gene promoter [17,24]. Several PRLR/JAK/STAT target genes are associated with the regulation of tumorigenesis, immunomodulation, and metastasis [9,24,25,26]. Of particular interest, the repair of double-strand DNA breaks and base lesions triggered by radiotherapy is strongly influenced by genes within the PRLR/JAK/STAT axis [27].

The association between PRLR and breastfeeding, treatment-targeted receptors, and JAK/STAT signaling suggests that *PRLR* variants could influence treatment response. However, to the best of our knowledge, no identified *PRLR* variants have been shown to differentially impact breast cancer prognosis across treatment modalities. Therefore, our primary aim was to investigate whether *PRLR* genetic variants could serve as treatment-predictive factors in a cohort of women diagnosed with primary breast cancer. Secondly, we examined the impact of *PRLR* on treatment-associated processes using a public database.

## 2. Materials and Methods

### 2.1. Patients

Patients in this study belonged to the prospective, population-based BC-Blood cohort in Sweden. Female patients with a first diagnosis of breast cancer who underwent surgery in 2002–2016 were included at Skåne University Hospital in Lund, Sweden. Before inclusion, all patients provided written informed consent. Upon inclusion, medical staff collected blood samples in EDTA tubes and body measurements. Patients filled out a three-page questionnaire addressing lifestyle factors, such as breastfeeding and smoking. For three years post-surgery, body measurements were taken regularly, follow-up questionnaires were completed, and patient medical charts were reviewed for information about tumors and adjuvant treatments. After three years, patients completed a questionnaire biannually, while clinical information was obtained from pathology reports and medical charts until the end of the follow-up on 30 June 2019.

**Figure 1:**
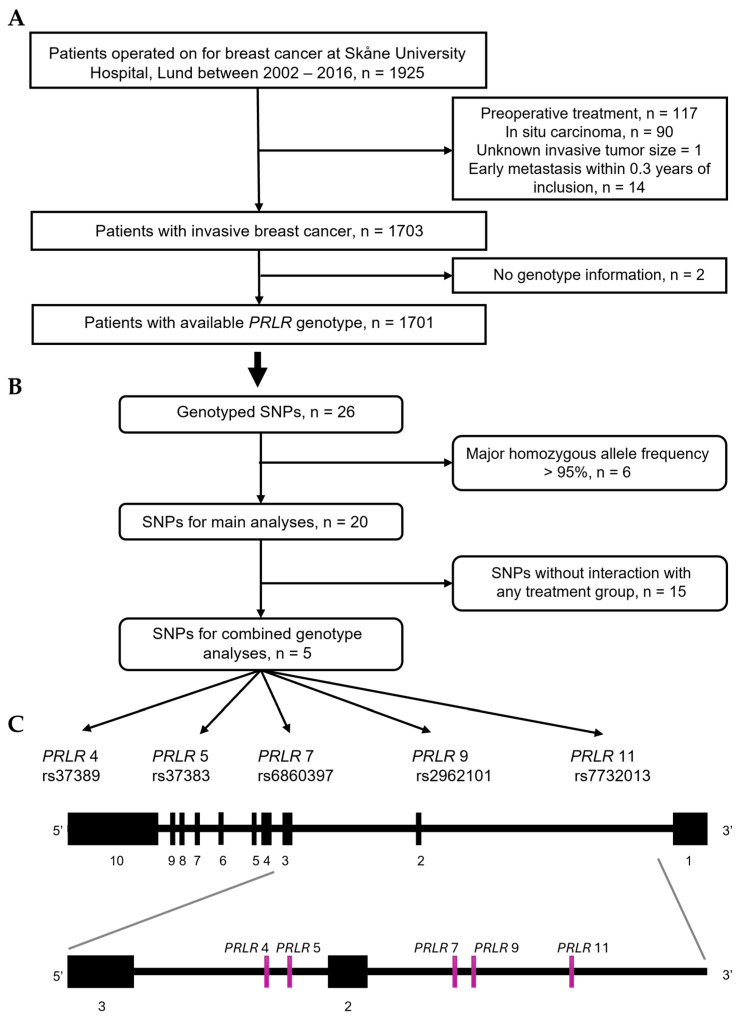
Flowchart of (**A**) patients participating in study, (**B**) PRLR genetic variants selected for main and additional analyses. (**C**) Schematic representation of five selected *PRLR* SNPs within *PRLR* gene.

Only patients with primary breast cancer, without a history of cancer in the past 10 years, were included in the study. Patients were excluded from the study if they (1) received pre-operative treatment, (2) had only in situ carcinoma, (3) presented with metastasis within 0.3 years of inclusion (Figure 1A). The BC-Blood cohort received ethical approval from the ethics committee at Lund University (Dnr 75-02, 37-08, 658-09, and amendments).

### 2.2. Genotyping of PRLR Single Nucleotide Polymorphisms

Collected EDTA blood samples were centrifuged and the plasma portions were removed. Buffy coat was mixed with red blood cells, and samples were frozen to −70 °C within a 2 h timeframe. Whole-blood DNA was extracted using the DNeasy Blood and Tissue kit (69504, Qiagen, Hilden, Germany) and processed with QiaCube (9002864, Qiagen, Hilden, Germany), following the manufacturer’s instructions. SNP genotyping was performed at the Center for Translational Genomics (CTG, Lund University) using OncoArray technology (WG-355, Illumina, San Diego, CA, USA). Genotype calling details are described elsewhere [28]. Standard quality control measures were implemented. Samples with low call rates (<1 × 10^−5^), *PRLR* SNPs with minor allele frequencies < 1%, or a call rate < 99% were excluded from the analyses.

Out of 26 *PRLR* SNPs that passed the quality control, 6 had homozygosity of the major genotype frequency > 95% and were excluded from the analyses (Figure 1B). The remaining 20 SNPs were utilized for subsequent analyses: PRLR 1 (rs387032), PRLR 2 (rs37389), PRLR 3 (rs37383), PRLR 4 (rs7734558), PRLR 5 (rs6860397), PRLR 6 (rs2962089), PRLR 7 (rs2962101), PRLR 8 (rs9292571), PRLR 9 (rs7732013), PRLR 10 (rs6451185), PRLR 11 (rs4703503), PRLR 12 (rs16872491), PRLR 13 (rs1039428), PRLR 14 (rs7720754), PRLR 15 (rs873456), PRLR 16 (rs7718468), PRLR 17 (rs4235652), PRLR 18 (rs1609500), PRLR 19 (rs6878684), and PRLR 20 (rs7735260).

PRLR 1 (rs387032) is located in the 3′ UTR region on exon 10, and the remaining *PRLR* SNPs are intronic. Only PRLR 15 (rs873456) and PRLR 18 (rs1609500) are in linkage disequilibrium (LD). For 13 *PRLR* SNPs (PRLR 1–3, 5–10, 12, 14, 17, 20), homozygous and heterozygous variants were merged due to genotype frequencies < 5%. The PRLR 7 genotype was imputed for one patient based on the most likely haplotype frequency according to the 1000Genome database, using the R package LDlinkR (v1.4.0), and used in all analyses [29]. The reference genotype was defined as the homozygous genotype with the highest frequency in the study population.

### 2.3. Variables

*PRLR* SNPs were analyzed in relation to patient and tumor characteristics. Age at inclusion was treated as a continuous variable and dichotomized at 50 years. Individuals below this threshold were considered pre-menopausal due to uncertainties about natural menstrual cycles among patients using exogenous hormones or those who had undergone hysterectomy without bilateral oophorectomy. Body Mass Index (BMI) was divided into <25 kg/m^2^, ≥25 kg/m^2^, and unknown. The number of children was categorized as nulliparous, 1–2 children, or ≥3 children. Age at first birth in parous patients was grouped into <20, 20–24, 25–29, and ≥30 years. Additionally, in parous patients, breastfeeding of the first child and total breastfeeding duration were dichotomized as 0–12 months and >12 months, based on prior findings [23]. To maintain sample size, a sublevel was added for unknowns (*n* = 16 and *n* = 12, respectively). In cases of twins, the first child referred to the twin who was breastfed the longest, and total breastfeeding duration only considered the first twin.

Pathological (invasive) tumor size (pT) was dichotomized as pT1 (<20 mm) and a combination of pT2, pT3, and pT4 (≥20 mm, or involving the skin or muscle regardless of size). Histological grade was dichotomized as grade III vs. I–II. ER and PgR status were dichotomized with a cut-off of 10% according to clinical routine in Sweden [5]. HER2 status was implemented into clinical routine from November 2005 [30] onward and was dichotomized as per Swedish guidelines as amplified or non-amplified [5]. HER2 gene amplification was determined by routine immunohistochemistry and in situ hybridization, and data was retrospectively supplemented with a dual gene protein assay [31]. Adjuvant treatments, including chemotherapy, trastuzumab, radiotherapy, and endocrine treatments (i.e., tamoxifen and aromatase inhibitors), were dichotomized into ever used and never used, regardless of duration or dose. Most patients received more than one type of adjuvant treatment.

The primary endpoint was any new breast cancer event, which included locoregional recurrence, contralateral breast cancer, or distant metastasis. The secondary endpoint was death due to any cause. Thirty-eight patients presented with bilateral tumors. Tumor characteristics and final surgical technique referring to the most aggressive side are used throughout the study.

### 2.4. Statistical Analyses

The breast-cancer-free interval (BCFI) was defined as the time between inclusion and the first breast cancer event. Patients without a breast cancer event before the end of follow-up were censored at the last completed follow-up before emigration or death due to any cause.

Univariable survival analyses for the 20 *PRLR* SNPs involved Log-rank tests and Kaplan–Meier curves using the R package survminer (v0.4.9) [32]. In the univariable analyses, the Log-rank test considered all new breast cancer events up to 30 June 2019. The Kaplan–Meier curves had a cut-off after 15.1 years, resulting in the exclusion of one event. Cox proportional hazard regression was used for multivariable survival analyses, utilizing the R package survival (v3.5-8) [33] to estimate the hazard ratio (HR) with a 95% confidence interval (CI) for BCFI.

A univariable model was utilized to explore the association between a genetic variant and BCFI. Multivariable models were constructed based on the univariable model, adjusting for age, tumor characteristics, adjuvant treatments, and BMI. The main multivariable model adjusted for age at inclusion (continuous), tumor size (pT2/3/4), any axillary lymph node metastasis, histological grade III, ER+, PgR+, chemotherapy, radiotherapy, tamoxifen, aromatase inhibitors, trastuzumab, and BMI. HER2 was not included, as testing was not adopted into clinical practice until November 2005 [31]. Interaction models incorporated the main multivariable model, an interaction term between the genotype, and one adjuvant treatment at a time.

The univariable, multivariable, and interaction models were applied individually to the 20 SNPs. Interactions between SNPs and radiotherapy on BCFI (*p* ≤ 0.1) were used to select five SNPs (PRLR 4, 5, 7, 9, and 11) and compile combined genotypes. Analyses were then repeated using the combined genotype variable, with the combination of all major alleles as reference.

Haplotypes of the five SNPs were analyzed to confirm the findings of the combined genotype analyses. The univariable, multivariable, and radiotherapy interaction analyses were repeated with categories defined by individual haplotype copy numbers (0, 1, or 2). Haplotypes were constructed either through cross-tabulations or by analyzing their frequency. For four patients with three genotype combinations, haplotype frequencies could not be accurately determined through cross-tabulations, instead the haplotypes were predicted based on the most probable haplotype combinations. Comparisons were made between haplotype frequencies and those of European populations in the 1000Genome database, and associations between variants and *PRLR* gene expression were examined with LDlinkR R package (v1.4.0) [29]. Frequencies of PRLR 4, 5, 7, 9, and 11 were similar to the 1000Genome haplotypes. Haplotypes with frequencies < 5% were grouped into a ‘rare’ haplotype category.

Interaction analyses between combined genotypes and radiotherapy were performed using the time between inclusion and death due to any cause to determine the interaction effect on overall survival. To assess the persistence of a radiotherapy interaction on BCFI, after accounting for the risk of death before any breast cancer event, competing risk analysis was performed. The Fine–Gray sub-distribution hazard model was used (cmprsk R package, v2.2-12) [34], with any new breast cancer event as the primary event and death due to any cause as the competing risk. Sensitivity analyses were performed by adjusting for either breastfeeding of the first child > 12 months or total breastfeeding duration > 12 months, separately.

The PS Power and Sample Size program (v.3.0, Vanderbilt University, Nashville, TN, USA) was utilized to perform power calculations for the detection of HR limits with at least 80% power and an α of 0.05 [35]. For the Swedish cohort, a 14-year accrual time and additional 3-year follow-up were considered. In single-SNP analyses, considering a median survival time of 5.1 years, with 1701 patients, 7% having the lowest-frequency variant and 57% having the highest-frequency variant, we would be able to detect true HRs of ≤0.74 or ≥1.40 and ≤0.85 or ≥1.18, respectively. For radiotherapy subgroup analysis, with 572 non-radiotherapy-treated patients, 5% of patients in the smallest combined genotype group, 32% of patients in the biggest combined genotype group, and considering median survival times of 5.1 years, we would be able to detect true HRs of ≤0.54 or ≥2.06 and ≤0.75 or ≥1.36, respectively. Lastly, with 1129 radiotherapy-treated patients, with 5% of patients in the smallest combined genotype group, 37% of patients the biggest combined genotype group, and considering median survival times of 7.0 and 5.1 years, we would be able to detect true HRs of ≤0.63 or ≥1.73 and ≤0.82 or ≥1.24, respectively. All *p*-values were two-tailed. In the individual SNP models, an α of 0.1 was considered to identify SNPs for further investigation. For models involving combined genotypes, including interaction and additional analyses, an α of 0.05 was used to determine significance. The number of tests (of combined genotypes or all combined haplotypes) informed the Bonferroni correction applied to the *p*-values.

### 2.5. Public Cohort Analyses

Patients from the publicly available METABRIC cohort (www.cbioportal.org accessed on 1 August 2024) were selected with matched clinical and mRNA expression data (*n* = 1980) [36,37]. The cohort was stratified into tertiles of *PRLR* expression: low (T1), intermediate (T2), and high (T3). Patient samples in each tertile were further divided into two treatment groups (those who received post-operative radiotherapy and those who did not) and further categorized by their respective BCFI (>5 or >10 years) (Supplementary Figure S2). Analysis of differentially expressed genes (DEGs) in T3 compared to T1 in each subgroup was performed with the limma R package (v3.60.4), applying a multivariable model adjusting for age at inclusion (continuous), tumor size, lymph node involvement, histological grade III, receptor status (ER+, PgR+, and HER2+), chemotherapy, endocrine therapy, surgery type, and cohort batch. Enrichment analyses of gene ontology (GO) biological process (BP) terms were performed with the ClusterProfiler R package (v4.12.2) [38] and illustrated with ggplot2 (v3.5.1). The enrichment of Reactome genesets was conducted with GSEA software (v4.3.3) [39]. The profiling of dominant carcinoma ecotypes (CEs) in each *PRLR* tertile was performed with EcoTyper (https://ecotyper.stanford.edu/carcinoma/ accessed on 2 December 2024) [40]. In preparation for this analysis, the METABRIC gene expression matrix was normalized (snmAnaly function, ExpressionNormalizationWorkflow R package, v1.34.0) using all but two variables involved in DEG analysis, excluding chemotherapy and hormone therapy. Normalized mRNA microarray intensities were then input into the EcoTyper tool “Analyze Bulk Expression Data”. Differences in major ecotype frequency between tertiles were statistically analyzed using the Chi-square test, and the number of CEs (*n* = 10) was used to apply a Bonferroni correction to *p*-values.

Transcriptional networks (TNs) were reconstructed using the RTN package (v2.32.0) [41], with the METABRIC data as input. Gene expression matrices of patient tumors were utilized after stratification according to radiotherapy status and further subdivision into four groups: (1) *PRLR*-low (T1) and no BC event within 5 years (BCFI > 5 years); (2) *PRLR*-low with BC event within 5 years (BCFI ≤ 5 years); (3) *PRLR*-high (T3) and BCFI > 5 years; (4) *PRLR*-high and BCFI ≤ 5 years. The RTN tool infers regulons, i.e. regulatory units comprising a transcription factor (TF) and its target genes, based on mutual information and multiple hypothesis testing. A list of all known human TFs (*n* = 1606) was obtained from the TF-Link gateway (https://tflink.net/accessed on 28 April 2025) [42] and included in the TN analysis. Bootstrap and Data Processing Inequality analyses were utilized to remove unstable TF–gene pairs (*n*Permutations = 1000) and redundant interactions, respectively. Conditional analysis [43] was then performed to interrogate *PRLR* as potential modulator of TF activity (*p* ≤ 0.001). To explore the main influenced pathways, gene symbols of the significantly and exclusively *PRLR*-modulated TFs in each subgroup were used as input for an overrepresentation analysis of PANTHER pathways (v19, pantherdb.org accessed on 9 May 2025) with Fisher’s exact test and an FDR correction cut-off ≤ 0.05. All significantly overrepresented pathways for each group were plotted (ggplot2 R package, v3.5.1). In parallel, Master Regulator Analysis (MRA) [44] was performed on the TN to reveal regulons with statistically significant (Benjamini–Hochberg-corrected *p* ≤ 0.05) overlap with genes overexpressed in each patient subgroup and plotted as a two-tailed GSEA.

## 3. Results

### 3.1. Clinicopathological Characteristics

Of the 1925 patients included in the BC-Blood study (2002–2016), 1701 met the inclusion criteria. A flowchart of the included patients is shown in Figure 1A. Patients were followed for up to 15 years until 30 June 2019, with a median follow-up of 5.1 years (interquartile range 3.1–9.1 years) for those still at risk (*n* = 1463). During follow-up, 238 patients experienced a new breast cancer event. A total of 218 patients died, 113 of whom had a previous breast cancer event. Within the cohort, 38% of the patients underwent mastectomy as the final surgical technique. Most patients received more than one modality of adjuvant treatment. Out of the 1701 patients, 29% received chemotherapy and 66% underwent radiotherapy. Of the 1504 patients with ER+ tumors, 59% were given tamoxifen and 46% received aromatase inhibitors. Additionally, 150 patients were diagnosed with HER2+ tumors after introducing HER2 testing in November 2005, and 79% of those patients received trastuzumab. Descriptive clinicopathological information for all 1701 patients is presented in Table 1.

### 3.2. PRLR Genotype Survival Analyses and Systemic Adjuvant Therapies

**Figure 2:**
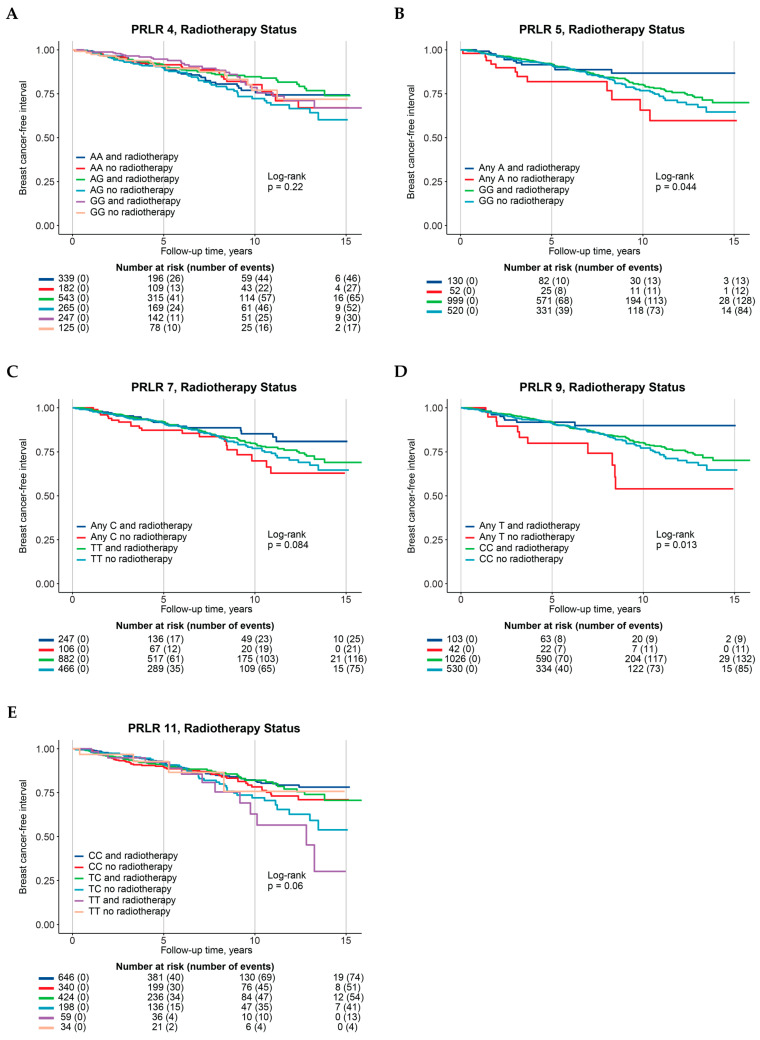
Kaplan–Meier curves, Log-rank *p*-values, and at-risk tables of association between *PRLR* genotypes with interaction with radiotherapy and breast-cancer-free interval, stratified according to radiotherapy groups. (**A**) PRLR 4, (**B**) PRLR 5, (**C**) PRLR 7, (**D**) PRLR 9, (**E**) PRLR 11.

The 20 genotyped *PRLR* SNPs that met the quality control thresholds (Figure 1B and Supplementary Table S1) were selected for additional statistical analyses to identify potential SNPs for future investigations (α = 0.1). Survival analyses using BCFI as the endpoint showed no associations between individual SNPs and BCFI, both in the univariable model (Figure 2) and in the full multivariable model adjusted for age (continuous), tumor characteristics, adjuvant treatment, and BMI (Supplementary Table S2A,B). Furthermore, introducing an interaction variable with one adjuvant treatment at a time revealed no interactions between *PRLR* SNPs and chemotherapy, tamoxifen (ER+ tumors), or aromatase inhibitors (ER+ tumors) (Supplementary Table S2C).

Multivariable interaction analysis involving radiotherapy revealed improvements in BCFI for five SNPs (Figure 1C) compared to their corresponding reference genotypes: PRLR 4 AG genotype (HR_int_ 0.56, 95% CI 0.31–1.03), PRLR 5 any A (HR_int_ 0.43, 95% CI 0.19–1.01), PRLR 7 any C (HR_int_ 0.52, 95% CI 0.27–1.00), and PRLR 9 any T (HR_int_ 0.33, 95% CI 0.13–0.84, Table 2 and Supplementary Table S2D). On the contrary, the minor TT-genotype of PRLR 11, when combined with radiotherapy, was linked with a marginally worse prognosis (HR_int_ 3.28, 95% CI 0.89–12.15) than the major CC-genotype (Table 2—single-SNP genotypes; Supplementary Table S2D).

### 3.3. Combined PRLR Genotype Survival Analyses and Adjuvant Radiotherapy

To further analyze the five SNPs (PRLR 4, 5, 7, 9, 11) with radiotherapy interaction tendencies, we constructed combined genotypes. Of all potential genotype combinations, 49 were present in the cohort. In total, 6 combinations had frequencies ≥ 5% and the remaining 43 combinations (35.7%) were grouped into a ‘rare’ genotype combination (Table 2—combined genotypes). The presence of any minor allele in PRLR 5, 7, or 9 resulted in a ‘rare’ combination. The combination of all major alleles (AA/GG/TT/CC/CC, *n* = 279) was used as the reference. Supplementary Table S3A,B present descriptive data on patient and tumor characteristics in relation to the five SNPs, and Supplementary Table S4A,B show information regarding combined genotypes.

The combined genotype 5 (AA/GG/TT/CC/TC) showed approximately a two-fold nominally significant increase in the incidence of new breast cancer events in both univariable and multivariable analyses (HR_adj_ 1.90, 95% CI 1.08–3.35), without interaction terms (Table 2 and Supplementary Table S5A). However, this outcome was not significant after implementing Bonferroni correction (*P*_adj_ = 0.2), and no other genotype combinations showed an association with BCFI.

Additionally, no interactions were found between combined genotypes and chemotherapy or tamoxifen on BCFI. However, a significant interaction was discovered between combined genotype 4 (GG/GG/TT/CC/TC) and aromatase inhibitors on BCFI (HR_int_ 0.14, 95% CI 0.02–0.76), although it was not significant after correction (*P*_adj_ = 0.1) (Supplementary Table S5B).

Combined genotype 2 (AG/GG/TT/CC/TC) and the ‘rare’ combination both exhibited significant interactions with radiotherapy on BCFI, maintaining significance even after Bonferroni correction. When compared to the reference, about a four-fold better prognosis (*P*_adj_ = 0.02) was observed for combined genotype 2 together with radiotherapy (HR_int_ 0.23, 95% CI 0.09–0.62). For the ‘rare’ combination, a three-fold improvement in prognosis (*P*_adj_ = 0.04) together with radiotherapy was observed (HR_int_ 0.29, 95% CI 0.12–0.71). Additionally, combined genotype 6 (GG/GG/TT/CC/CC) demonstrated a significant interaction with radiotherapy (HR_int_ 0.22, 95% CI 0.05–0.89), although this was not significant after correction (*P*_adj_ = 0.2) (Table 2—combined genotypes; Supplementary Table S5C). The Kaplan–Meier curves representing the interaction between combined genotypes and radiotherapy on BCFI are presented in Supplementary Figure S1.

Given the observed interactions, patients were then stratified based on radiotherapy status (treated or not). Patients who did not receive radiotherapy and carried genotype 2 exhibited a significantly worse prognosis compared to the reference (HR_int_ 2.79, 95% CI 1.27–6.14) (Table 3). On the other hand, radiotherapy-treated patients carrying genotype 2 displayed a similar, albeit slightly improved, prognosis compared to the reference (HR_int_ 0.63, 95% CI 0.35–1.15). Comparable results were observed for genotype combinations 6 and ‘rare’ (Table 3).

Next, haplotype analyses were performed with the reference defined as carrying two copies of a haplotype (Supplementary Table S6A). The other categories included having one copy or not having any copies. There was a significant interaction between radiotherapy and the absence of AGTCC copies, which was no longer significant after statistical correction (HR_int_ 0.35, 95% CI 0.14–0.87). However, the interaction between radiotherapy and the presence of one AGTCC copy remained significant on BCFI even after correction (HR_int_ 0.30, 95% CI 0.13–0.71). These findings corroborate our combined genotype results, as combined genotype 2 possesses one AGTCC haplotype.

In summary, patients with any minor allele who were not treated with radiotherapy had a higher incidence of breast cancer events than those with all major alleles, whereas the opposite was observed in most patients with minor alleles who had been treated with radiotherapy. These findings suggest that radiotherapy efficiency is impacted by *PRLR* genotypes, with greater benefits in patients with minor alleles.

### 3.4. Competing Risk and Sensitivity Analyses

To investigate if death could be a confounding factor, combined genotype interaction analyses for radiotherapy on overall survival were conducted, and no significant associations or interactions were found (Supplementary Table S6B). In addition, we performed competing risk analyses with new breast cancer events as the main endpoint and death due to any cause as the competing event to calculate the sub-hazard ratios for interaction with radiotherapy. The risk estimates remained essentially the same, as displayed in Supplementary Table S6C.

Furthermore, since indications of potential confounding by breastfeeding were observed in the descriptive statistics conducted by individual SNP genotype (Supplementary Table S3A), sensitivity analyses were performed to determine whether our results may be explained by breastfeeding duration. In these analyses, breastfeeding of the first child for >12 months and a total breastfeeding period >12 months were individually included as covariates in two separate interaction models with radiotherapy. For both the individual analyses and combined genotype analyses, adjusting for breastfeeding did not substantially alter the results (Supplementary Table S6D).

### 3.5. PRLR Expression and Radiotherapy Response in METABRIC Cohort

To further interrogate the potential effects of the identified SNPs with radiotherapy interactions on BCFI, the LDlinkR R package (v1.4.0) was utilized. Analysis of the SNPs in the LDlink high-coverage database [45] revealed that the minor alleles of SNPs 4, 7, and 9 were linked with proxy *PRLR* variants associated with lower *PRLR* transcript abundance in Expression Quantitative Trait Loci (eQTL) results (Supplementary Table S7). For PRLR 5, the linked alleles did not show any association with *PRLR* expression. In contrast, PRLR 11 proxy variants were associated with a predicted increase in *PRLR* expression in vascular tissue.

Considering the potential effects of the PRLR/JAK/STAT axis on the efficacy of radiotherapy, we analyzed the BCFI of METABRIC patients in the context of *PRLR* expression tertiles (T3 vs. T1) and their response to radiotherapy (Supplementary Figure S2A). Supplementary Table S8A presents descriptive information on the characteristics of the METABRIC population.

**Figure 3:**
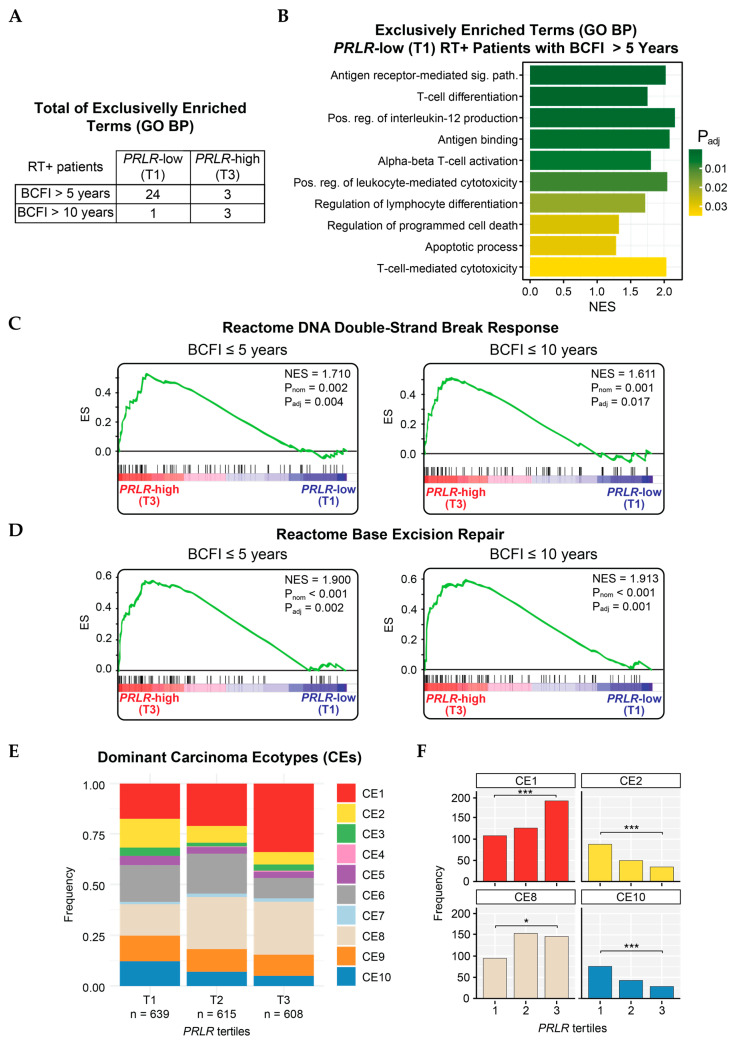
Enriched pathways in METABRIC patients. (**A**) Total of GO biological process terms exclusively enriched in groups of radiotherapy-treated patients stratified according to breast-cancer-free interval (over either 5 or 10 years) and PRLR expression (low or high). (**B**) Presentation of 10 out of 24 pathways enriched in radiotherapy-treated patients with low-PRLR (T1) tumors who did not present breast cancer event within 5 years (BCFI > 5 years). Sig. path = signaling pathway; Pos. reg = positive regulation; NES = Normalized Enrichment Score. *P*_adj_ = adjusted *p*-value. (**C,D**) GSEA curves displaying enrichment of Reactome-sourced pathways: (**C**) DNA double-strand break response and (**D**) base excision repair in radiotherapy-treated patients with PRLR-high (T3) tumors and breast cancer event within 5 (left) or 10 years (right). (**E**) Visualization of dominant carcinoma ecotypes (CEs) in METABRIC samples stratified by PRLR tertiles. (**F**) Frequencies of samples with CE1, CE2, CE8, and CE10 as dominant ecotypes per PRLR tertile. *** = *P*_adj_ (Chi-square test between T1 and T3) < 0.001. * = *P*_adj_ (Chi-square test between T1 and T3) < 0.05.

The analysis of the enriched biological processes of differentially expressed genes in *PRLR*-low tumors from radiotherapy-treated patients without a breast cancer event within 5 years (BCFI > 5 years, *n* = 262) showed the significant enrichment (P_adj_ < 0.05) of pathways indicative of increased immune activity. This was compared to (1) those with *PRLR*-high tumors, (2) those who experienced a breast cancer event, (3) those who did not receive radiotherapy (Figure 3A,B and Figure S2B, Supplementary Table S8B). However, immuno-related pathways were no longer significantly enriched by 10 years. Moreover, *PRLR*-high tumors in patients who were treated with radiotherapy and experienced a breast cancer event within 5 or 10 years displayed the significant enrichment of DNA double-strand break responses (Normalized Enrichment Score (NES) BFCI ≤ 5 years= 1.71, *P*_adj_ = 0.004; NES BFCI ≤ 10 years= 1.61, *P*_adj_ = 0.017) and base excision repair pathways (NES BFCI ≤ 5 years= 1.90, *P*_adj_ = 0.002; NES BFCI ≤10years = 1.91, *P*_adj_ = 0.001). This was compared to radiotherapy-treated patients with *PRLR*-low tumors (Figure 3C,D).

Analysis of tumor tissue composition, based on the EcoTyper deconvolution tool, revealed that *PRLR*-high tumors (T3) had an abundant representation of the CE1 signature compared to *PRLR*-low tumors (T1, *P*_adj_ < 0.001) (Figure 3E,F). This suggests an enrichment of epithelial-to-mesenchymal transitioning (EMT), TGFβ signaling, angiogenesis, and a deficiency in lymphocytes within this group, which are characteristics of aggressive tumors. CE8 was also significantly overrepresented in the high-*PRLR* group; however, the biological impact of this signature requires further exploration. Conversely, samples with low *PRLR* expression (T1) displayed a significantly increased CE2 signature, which signifies an enrichment of wound-healing processes and copy-number variations (CNVs). Interestingly, the pro-inflammatory CE10 was also overrepresented in *PRLR*-low as compared to *PRLR*-high tumors (*P*_adj_ < 0.001) (Figure 3E,F), indicating a higher IFNγ-mediated response, increased lymphocyte infiltration, and apoptosis.

**Figure 4:**
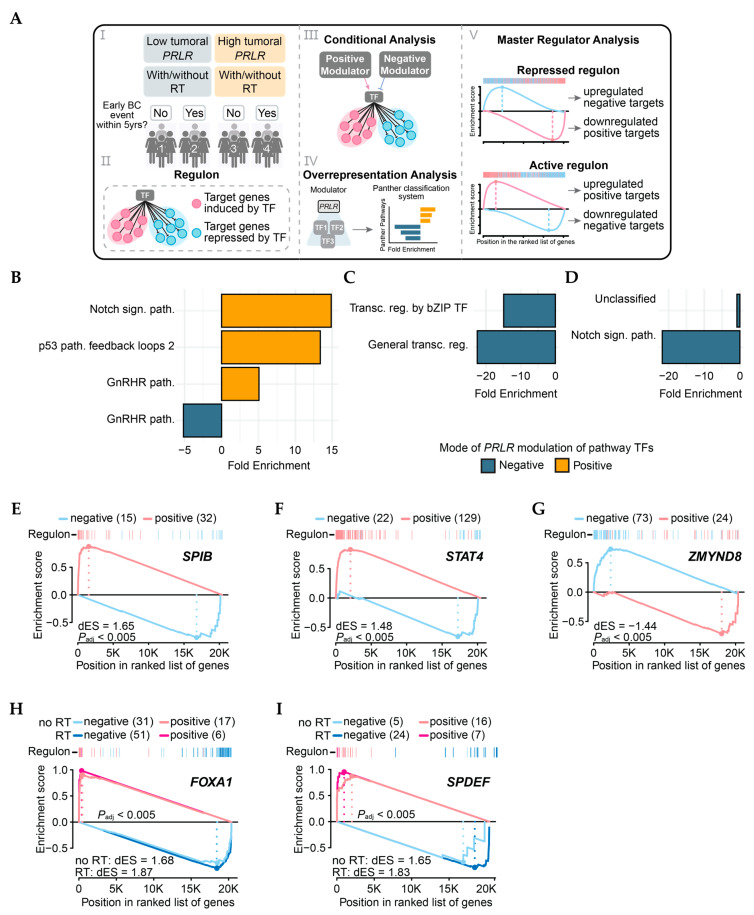
Transcriptional analysis of METABRIC samples. (**A**) I: Patient groups utilized for TN reconstruction. II: Representation of regulon—regulatory unit comprising TF and its target genes. III: Illustration of two possible results of conditional analysis, where PRLR can modulate regulon either positively or negatively. IV: Overrepresentation analysis performed with gene symbols of TFs significantly modulated by PRLR as input. V: Schematics of master regulator status where repressed regulons present with overexpression of target genes repressed by regulating TF and downregulation of positive TF targets. Active regulons present opposite scenario. (**B**–**D**) Results of overrepresentation analysis of PANTHER pathways in RT+ groups compared to no RT in following groups of patients: (**B**) PRLR-low tumors and no BC events within 5 years; (**C**) PRLR-low tumors and BC events within 5 years; (**D**) PRLR-high tumors and no BC events within 5 years. sign. = signaling; path. = pathway; transcr = transcriptional; reg. = regulation. (**E**–**G**) Master regulators of PRLR-low tumors in radiotherapy-treated patients without early events within 5 years. (**H**,**I**) Master regulators of PRLR-high tumors in patients (treated or not with radiotherapy) who experienced early BC events within 5 years. dES = difference in Enrichment Score (quantification of regulon activity).

To assess PRLR impact with yet another level of information, transcriptional networks were reconstructed for four subgroups of radiotherapy-treated and non-radiotherapy-treated patients (with high (T3) or low (T1) tumoral *PRLR* and BCFI≤ or >5 years, Figure 4A) using the RTN R package (v2.32.0). Regulons were inferred for all 1606 known human transcription factors (TFs), followed by further analyses (Figure 4A). Conditional analysis revealed that *PRLR* could modulate (positively or negatively) the activity of 819 TFs in total (Supplementary Table S9). In patients with *PRLR*-low tumor expression and without an early BC event, pathway analysis of TFs exclusively modulated by *PRLR* in this group showed that *PRLR* significantly induced TFs of the Notch and p53 pathways (Figure 4B). For patients who experienced an early BC event despite having *PRLR*-low tumors, *PRLR* was a negative modulator of TFs from the bZIP pathway and general transcription (Figure 4C), and it also suppressed the Notch signaling pathway in the *PRLR*-high group without early events (Figure 4D). Despite 61 regulons having their activity significantly modulated by *PRLR* in the fourth group of patients (*PRLR*-high with an early BC event, see Supplementary Table S9), no specific pathways were significantly induced or repressed. Analysis of master regulators (MRs, Supplementary Table S10) revealed that radiotherapy-treated patients who did not have an early event and had *PRLR*-low tumors displayed the particular activation of regulons responsible for lymphocyte and cytokine modulation (e.g., *SPIB* and *STAT4*, dES = 1.65 and 1.48, respectively, *P*_adj_ < 0.005—Figure 4E,F) when compared to analogous non-radiotherapy-treated patients. Furthermore, the *ZMYND8* regulon, known for promoting breast cancer aggressiveness, was found to be suppressed in this group of patients (dES = −1.44, *P*_adj_ < 0.005—Figure 4G). For those with *PRLR*-high tumors and with an early BC event, *FOXA1* (no RT dES = 1.68; RT dES = 1.87, *P*_adj_ < 0.05—Figure 4H) and *SPDEF* (no RT dES = 1.65; RT dES = 1.83, *P*_adj_ < 0.05—Figure 4G), regulons were found to be activated regardless of radiotherapy status.

In summary, radiotherapy-treated patients in the METABRIC cohort who had *PRLR*-low tumors—representing the potential effect of three *PRLR* SNPs found in this study—pointed towards the better activation of immune pathways both at gene expression and transcriptional levels when compared to groups of patients with either *PRLR*-high tumors and/or BCFI ≤ 5 years, and/or no radiotherapy. Meanwhile, *PRLR*-high groups displayed the activation of treatment resistance pathways such as improved DNA repair at the gene expression level and the transcriptional activation of potential pro-tumorigenic cascades.

## 4. Discussion

The results of this study suggest that certain *PRLR* genotypes potentially affect radiotherapy efficacy. Patients who carry minor alleles in any of the following four *PRLR* SNPs (rs7734558, rs6860397, rs2962101, and rs7732013) may experience greater radiotherapy efficacy than those with all major alleles. Moreover, carriers of the most common combined genotype or ‘rare’ combinations had a poorer prognosis without radiotherapy than patients with all major alleles. However, the prognosis changed drastically for those who received radiotherapy. Given that the goal of radiotherapy is to prevent locoregional recurrence, our results suggest that patients with the combined genotype 2 (AG/GG/TT/CC/TC) or ‘rare’ combinations would derive extra benefits from radiotherapy compared to carriers of major alleles (AA/GG/TT/CC/CC). Similar results were found for other combinations of minor alleles, and these results were not confounded by the duration of breastfeeding. Interestingly, no interaction with radiotherapy was detected when using overall survival (death due to any cause) as the endpoint rather than BCFI. This finding further strengthens the idea that *PRLR* genotypes mainly impact radiotherapy efficacy rather than interacting with other systemic therapies. Furthermore, the higher incidence of new breast cancer events without an increased mortality rate is likely driven by late locoregional recurrences, which lead to lower mortality than distant metastases [46,47,48].

In breast cancer treatment, primarily healthy tissue is targeted with radiation to eliminate remaining tumor cells post-surgery. The cells adjacent to the tumor are likely to share genotypes with the genomic DNA used in this study. Additionally, our findings primarily rely on intronic SNPs, suggesting that these variants could (1) be in linkage with functional SNPs (tagSNPs), (2) have effect through alternative splice variants, (3) alter the binding or function of non-coding regulatory RNAs [49]. In a multiethnic cohort, the minor C-allele of PRLR 7 (rs2962101) was found to be linked to the minor allele of rs37364, leading to decreased PRL levels [50]. However, this linkage was not found in a Polish study, which might more closely resemble the Swedish cohort than the multiethnic cohort [51]. As the BC-Blood cohort lacked data on *PRLR* gene expression or protein levels of patients, we could not directly verify the association between genetic variants and PRLR levels in the same cohort.

Biologically, the eQTL-predicted reduction in *PRLR* expression linked with SNPs 4, 7, and 9 might reduce the activation of the JAK/STAT pathway, an indispensable signaling cascade for DNA repair and immune regulation [26]. A reduction in DNA repair capacity, especially for radiotherapy-induced DNA damage such as double-strand breaks and base lesions, could enhance radiotherapy efficacy [9,27]. Interestingly, when examining METABRIC gene expression data, radiotherapy-treated patients with poor prognosis and highly *PRLR*-expressing tumors showed the significant enrichment of pathways associated with enhanced DNA repair mechanisms. Also, the EcoTyper tool identified a pro-tumorigenic signature (CE1) enriched for TGFβ signaling, known for fostering radioresistance, in *PRLR*-high tumors [10]. However, such enrichment of radiotherapy-rescuing cascades was not directly observed at transcriptional level in the same group. In general, transcriptional analysis revealed activation of the *FOXA1* and *SPDEF* regulons in *PRLR*-high samples, both of which act as oncogenes in luminal subtypes [52,53]. It is worth noting that, although the METABRIC data was normalized to account for subtype differences, most of the cohort samples are classified as luminal. In a study of subtype-specific functions of *SPDEF* in breast cancer, this TF was seen to associate with DNA repair and TGFβ signaling [53]. Moreover, in non-small-cell lung cancer samples, *SPDEF* was shown to deplete the expression of *WDR11-DT*, a radiosensitizing long non-coding RNA that suppresses base excision and homologous recombination DNA repair mechanisms [54].

In radiotherapy-treated patients with good prognosis and *PRLR*-low tumors, a strong enrichment of anti-tumoral pathways (e.g., pro-inflammatory signatures, T-cell activation, and cytotoxicity) was observed in overrepresentation and ecotype explorations. Transcriptional analysis displayed the activation of the *SPIB* and *STAT4* regulons, as well as the repression of the *ZMYND8* regulon, as the main differential regulators in this group. The roles of SPIB and STAT4 in breast cancer, while not fully elucidated, were both associated with improved immune cell activity and better overall survival in previous studies [55,56,57]. On the other hand, the *ZMYND8* TF is known to promote breast cancer progression through stem cell modulation conferring therapeutic resistance and protecting it from oxidative stress [58,59,60]. Since radiotherapy induces the production of reactive oxygen species, the repression of *ZMYND8* as seen to happen in this subgroup of patients could lead to an improved radiotherapy response. Therefore, impaired tumor cell recovery post-radiotherapy, along with increased antigen presentation, might result in elevated tumor cell death rates and fewer breast cancer events for these patients [10].

The PRLR cascade may enhance radiotherapy efficacy via JAK/STAT signaling through the suppression of DNA repair. Previous studies demonstrate that both the genetic knockdown and pharmacological inhibition of STAT5A/B partially suppresses DNA repair, thereby augmenting radiotherapy efficacy in prostate cancer cell lines [27]. In addition, PRLR dimers interface with HER2 as a component of JAK/STAT signaling, which triggers the ERα pathway, further regulating tumorigenesis and metastasis [25]. The ERα pathway participates in the regulation of *PRLR* expression and can be activated by STAT5 [25]. These findings suggest that PRLR may affect the response to multiple breast cancer treatments, such as endocrine treatment, trastuzumab, and radiotherapy. However, in this study, *PRLR* genotypes were consistently associated with differential prognosis only in relation to radiotherapy-treated patients.

Nowadays, an increasing number of patients undergo breast-conserving surgery accompanied by radiotherapy. Interestingly, a recent study involving 48,986 women revealed that this combination of breast-conserving surgery and radiotherapy resulted in better survival rates than mastectomy, regardless of radiotherapy [61]. The heightened utilization of radiotherapy in breast cancer treatment could accentuate the relevance of our findings if validated. Clinically, there exists a need for more precise identification of (1) patients who require and respond to radiotherapy, (2) patients who can safely forego radiotherapy despite undergoing breast-conserving surgery, (3) patients for whom radiotherapy is non-beneficial and who would gain more from a different treatment modality. Consequently, the further validation of the findings of this study in independent cohorts is warranted as the authors were not able to gain access to such cohorts outside Sweden. A promising avenue of future studies could involve the relationship between *PRLR* genotypes, protein levels, tumor subtypes, and types of breast cancer events. Moreover, the potential role of PRLR inhibition as a therapeutic strategy for breast cancer warrants further exploration. This is reinforced by clinical trials with PRLR-targeted antibody–drug conjugates (ADCs), which have demonstrated good tolerability in human subjects and exhibited cytotoxic effects in preclinical models outside the context of radiotherapy [62,63].

This study is not without its limitations. Firstly, the selection of cohort participants may introduce bias due to language barriers, potentially excluding certain individuals. However, the main reason for non-inclusion was due to the limited availability of research nurses, and about 5% of patients did not have a definitive cancer diagnoses at the pre-operative visit and were not invited to participate [64]. Furthermore, the absence of ethnic background details for patients prevents the exploration of ethnic representation, although the majority of the patients in this population-based Swedish cohort were of European descent. Another limitation pertains to treatment. Patients who received neoadjuvant interventions were not included in our research due to the challenges of adjusting for tumor characteristics, potentially resulting in an underrepresentation of aggressive tumors. Finally, since this is a ‘real-world’ cohort, the treatment groups were not randomized. The patients with or without radiotherapy are not directly comparable, as tumor aggressiveness and type of surgery are deciding factors in the administration of radiotherapy. Patients with aggressive tumors and those who underwent breast-conserving surgery are more likely to receive radiotherapy [5]. Although 35–40% of the cohort patients underwent a mastectomy, this did not explain the different responses to radiotherapy among combined genotypes. Additionally, no significant differences were observed between the subgroups of combined genotype and surgery type.

## 5. Conclusions

We observed significant interactions between specific combinations of *PRLR* SNPs (AG/GG/TT/CC/TC) and radiotherapy in relation to clinical outcomes in a population-based cohort of women diagnosed with breast cancer. The minor alleles of three out of these five SNPs were associated with decreased *PRLR* expression, which could lead to diminished *PRLR* transcription. This decrease in *PRLR* transcription might contribute to the suppression of radiation-induced DNA damage repair and the enhancement of anti-tumoral immune activation, increasing the efficacy of radiotherapy. The regulons *SPIB*, *STAT4*, and *ZMYND8* were seen as the master regulators of immune modulation in *PRLR*-low patients who did not experience early breast cancer events. High *PRLR* levels (genetically predicted or measured via gene expression) indicate the priming of cancer cells towards radioresistance, but further explorations are required. Our findings suggest that, if validated, patient *PRLR* genotypes and transcriptional states could serve as predictive pharmacogenomics biomarkers for radiotherapy treatment. The topic of PRLR and radiotherapy merits further study.

## Figures and Tables

**Table 1:** Clinicopathological characteristics of 1701 included patients.

Characteristics	All Patients	Missing
	n = 1701 (%)	
Age at inclusion (years) ^a^	62 (52, 69)	0
Age ≥ 50 (years)	1367 (80)	0
BMI ≥ 25 (kg/m^2^)	836 (52)	101
Preoperative smoker, yes	300 (18)	7
Oral contraceptive use, ever	1239 (73)	3
MHT, ever	671 (40)	6
Children		1
Nulliparous	192 (11)	
1–2	1049 (62)	
≥3	459 (27)	
Age at first birth (years) ^b^		7
<20	188 (13)	
20–24	533 (35)	
25–29	502 (33)	
≥30	279 (19)	
Breastfeeding first child (months)		16
Nulliparous	192 (11)	
0–12	1440 (85)	
>12	53 (3.1)	
Breastfeeding total (months)		12
Nulliparous	192 (11)	
0–12	941 (56)	
>12	556 (33)	
Excessive milk production ^b^	230 (16)	75
Invasive tumor size pT2/3/4	441 (26)	0
Any axillary lymph node involvement	579 (34)	2
ER+	1504 (89)	2
PgR+	1214 (71)	2
HER2+	175 (11)	66
TNBC	129 (7.6)	8
Main histological type		0
No specific type	1366 (80)	
Lobular	194 (11)	
Mixed	141 (8.3)	
Histological grade III	470 (28)	6
Final surgical technique		
Mastectomy, yes	647 (38)	0
Adjuvant treatments		
Chemotherapy	488 (29)	0
Radiotherapy	1129 (66)	0
HER2+ as of Nov. 2005	n = 150	
Trastuzumab	118 (79)	0
ER+ tumors only	n = 1504	
Tamoxifen	892 (59)	0
Aromatase inhibitors	688 (46)	0

^a^ Median age (interquartile range). ^b^ In parous patients. BMI = Body Mass Index. MHT = menopausal hormone treatment. ER+ = estrogen-receptor-positive; PgR+ = progesterone-receptor-positive; HER2+ = human epidermal growth factor-positive; TNBC = triple-negative breast cancer.

**Table 2:** Effect estimates showing associations between BCFI and *PRLR* genotypes and genotype–radiotherapy interactions.

Genotype		Total (n)	Any BC Event (n)	Multivariable Model ^1^ (Without Interaction Term)			Interaction Radiotherapy ^2^		
				HR (CI 95%)	*P*_nom_	*P*_adj_	HR (CI 95%)	*P*_nom_	*P*_adj_
Single-SNP genotypes									
PRLR 4	AA	521	73	Ref	Ref	Ref	Ref	Ref	Ref
	AG	808	117	1.05 (0.78–1.41)	>0.3	1	0.56 (0.31–1.03)	0.06	1
	GG	372	48	0.94 (0.65–1.36)	>0.3	1	0.84 (0.39–1.79)	>0.3	1
PRLR 5	GG	1519	213	Ref	Ref	Ref	Ref	Ref	Ref
	Any A	182	25	0.95 (0.63–1.45)	>0.3	1	0.43 (0.19–1.01)	0.05	1
PRLR 7	TT	1348	192	Ref	Ref	Ref	Ref	Ref	Ref
	Any C	353	46	0.90 (0.65–1.25)	>0.3	1	0.52 (0.27–1.00)	0.05	1
PRLR 9	CC	1556	218	Ref	Ref	Ref	Ref	Ref	Ref
	Any T	145	20	1.05 (0.66–1.67)	>0.3	1	0.33 (0.13–0.84)	0.02	0.5
PRLR 11	CC	986	125	Ref	Ref	Ref	Ref	Ref	Ref
	TC	622	96	1.19 (0.91–1.55)	0.2	1	0.78 (0.45–1.36)	>0.3	1
	TT	93	17	1.63 (0.96–2.77)	0.07	1	3.28 (0.89–12.15)	0.07	1
Combined genotypes									
1. AA/GG/TT/CC/CC		279	33	Ref	Ref	Ref	Ref	Ref	Ref
2. AG/GG/TT/CC/TC		285	41	1.21 (0.76–1.91)	>0.3	1	0.23 (0.09–0.62)	0.003	0.02
3. AG/GG/TT/CC/CC		256	33	1.21 (0.75–1.97)	>0.3	1	0.48 (0.17–1.36)	0.17	1
4. GG/GG/TT/CC/TC		95	17	1.49 (0.81–2.73)	0.2	1	0.55 (0.15–1.98)	>0.3	1
5. AA/GG/TT/CC/TC		91	20	1.90 (1.08–3.35)	0.025	0.15	0.50 (0.15–1.70)	0.27	1
6. GG/GG/TT/CC/CC		87	11	1.06 (0.53–2.11)	>0.3	1	0.22 (0.05–0.89)	0.03	0.2
7 Rare		608	83	1.18 (0.78–1.77)	>0.3	1	0.29 (0.12–0.71)	0.0065	0.039

^1^ Cox proportional hazard regression adjusted for age at inclusion, tumor characteristics, BMI, and adjuvant treatments. ^2^ Effect size of interaction between genotypes and radiotherapy. Adjusted Cox regression with interaction term added. Ref = reference; HR = hazard ratio; CI = confidence interval; *P_nom_* = nominal *p*-value; *P_adj_* = Bonferroni-corrected *p*-value.

**Table 3:** Effect estimates showing associations between breast-cancer-free interval and *PRLR* genotypes, stratified by radiotherapy.

Combined Genotypes		Subgroup: No Radiotherapy ^1^					Subgroup: With Radiotherapy ^1^				
		Total (n)	Any BC Event (n)	HR (CI 95%)	*P*_nom_	*P*_adj_	Total (n)	Any BC Event (n)	HR (CI 95%)	*P*_nom_	*P*_adj_
1	AA/GG/TT/CC/CC	104	9	Ref	Ref	Ref	175	24	Ref	Ref	Ref
2	AG/GG/TT/CC/TC	94	21	2.79 (1.27–6.14)	0.01	0.06	191	20	0.63 (0.35–1.15)	0.1	0.8
3	AG/GG/TT/CC/CC	95	13	1.77 (0.75–4.18)	0.2	1	161	20	0.89 (0.49–1.63)	>0.3	1
4	GG/GG/TT/CC/TC	34	6	2.28 (0.79–6.62)	0.1	0.7	61	11	1.13 (0.53–2.38)	>0.3	1
5	AA/GG/TT/CC/TC	31	7	2.91 (1.06–7.99)	0.04	0.2	60	13	1.36 (0.67–2.78)	>0.3	1
6	GG/GG/TT/CC/CC	28	5	2.56 (0.85–7.75)	0.09	0.57	59	6	0.54 (0.22–1.34)	0.18	1
7	Rare	186	35	2.43 (1.16–5.11)	0.02	0.1	422	48	0.69 (0.42–1.15)	0.15	0.9

^1^ Cox proportional hazard regression adjusted for age at inclusion, tumor characteristics, BMI, and adjuvant treatments, stratified according to radiotherapy groups. Ref = reference; HR = hazard ratio; CI = confidence interval; *P*_nom_ = nominal *p*-value; *P*_adj_ = Bonferroni-corrected *p*-value.

## Data Availability

BC-Blood data are not publicly available due to privacy laws to protect patient confidentiality. Questions regarding data can be directed to the corresponding author (H.J.). The data of the METABRIC cohort are accessible from Curtis et al. (2012) [36] and Pereira et al. (2016) [37], as well as being available at cBioPortal. Bioinformatics and statistical analysis author’s contact information: kelin.goncalves_de_oliveira@med.lu.se.

## Supplementary Materials

The following supporting information can be downloaded at: https://www.mdpi.com/article/10.3390/cancers17142378/s1.
