# Supplementary material for: Prognostic Differences of Adjuvant Radiotherapy in Breast Cancer Cohorts Based on PRLR Genotypes, Expression, and Transcriptional Network Regulation
Source: Cancers (Basel). 2025 Jul 17;17(14):2378. doi: 10.3390/cancers17142378 (PMC12293342; doi:10.3390/cancers17142378)

Prognostic differences of adjuvant radiotherapy in breast cancer cohorts based on PRLR genotypes, expression, and transcriptional network regulation

Floor Munnik MSc[1], Kelin Gonçalves de Oliveira PhD[1], Christopher Godina MD, PhD[1], Karolin Isaksson MD, PhD[2] and Helena Jernström PhD[1,*]

[1] Department of Clinical Sciences Lund, Oncology, Lund University Cancer Center/Kamprad, Lund University and Skåne University Hospital, Barngatan 4, SE-221 85, Lund, Sweden
[2] Division of Surgery, Department of Clinical Sciences in Lund, Lund University and Department of Surgery, Skåne University Hospital, Kristianstad, Sweden.
* Correspondence: helena.jernstrom@med.lu.se; Tel.: +46 46177619

Table of Contents

Supplementary Figure Legends…………………...……………………..…………………………1

Supplementary Table Legends……………...……………………………………………………..1-3

Supplementary Figure S1………………….....…………………………………………….………... 4

Supplementary Figure S2…………………………………………………………………………… 5

OBS: Supplementary Tables are provided as .xls file.

Supplementary figure legends

Supplementary Figure S1. Kaplan-Meier curves, Log-rank *P*-values, and at-risk tables of the association between combined genotypes and breast cancer-free interval, stratified by radiotherapy. In the figure legend, the combined genotypes are numbered 1 – 7 from top to bottom. The 'rare' genotype consists of all combinations with a frequency < 5%. (A) Without radiotherapy (n = 572), (B) With radiotherapy (n = 1129).

Supplementary Figure S2. (**A**) Flowchart of patients included in the METABRIC cohort and patient numbers in each subcategory of *PRLR* tertiles, radiotherapy, and breast cancer event. (**B**) Venn-Diagram of enriched GO biological processes in METABRIC patients with or without breast cancer event in 5 years. Venn diagrams were built using InteractiVenn - Interactive Venn Diagrams (https://www.interactivenn.net/).

Supplementary table legends

Supplementary Table S1. (**A**) PRLR SNPs included in the study with corresponding chromosome:base pair coordinates.

(GRCh37) and allele frequencies.

Supplementary Table S2. (**A**) Univariable model results of individual *PRLR* SNPs estimates on BCFI. (**B**) Multivariable model results of individual *PRLR* SNPs estimates on BCFI. Model adjusts for age at inclusion, BMI, tumour characteristics and adjuvant treatments. (**C**) Interaction estimates between *PRLR* SNPs with the following adjuvant treatments: Chemotherapy, Tamoxifen, and Aromatase Inhibitor, on BCFI as endpoint. Model adjusts for age at inclusion, BMI, tumour characteristics and adjuvant treatments. (**D**) Interaction estimates between *PRLR* SNPs with Radiotherapy, on BCFI as endpoint. Model adjusts for age at inclusion, BMI, tumour characteristics and adjuvant treatments. Ref = reference; HR = Hazard ratio; CI = confidence interval

Supplementary Table S3. (**A**) Patient characteristics for all 1701 patients and by *PRLR* genotype of the five *PRLR* SNPs chosen for combined genotype analyses. [a] Median (Inter Quartile Range). BMI = Body Mass Index; MHT = menopausal hormone treatment. [b] In parous patients. (**B**) Tumour characteristics for all 1701 patients and by *PRLR* genotype of the five *PRLR* SNPs chosen for combined genotype analyses. ER+ = oestrogen receptor positive; PgR+ = progesterone receptor positive; HER2+ = human epidermal growth factor positive; TNBC = triple- negative breast cancer.

Supplementary Table S4. (**A**) Patient characteristics for all 1701 patients and by *PRLR* combined genotypes. [a] Median (Inter Quartile Range). BMI = Body Mass Index; MHT = menopausal hormone treatment. [b] In parous patients. (**B**) Tumour characteristics for all 1701 patients and by *PRLR* combined genotypes. ER+ = oestrogen receptor positive; PgR+ = progesterone receptor positive; HER2+ = human epidermal growth factor positive; TNBC = triple-negative breast cancer.

Supplementary Table S5. (**A**) Univariable model results of combined genotypes estimates on BCFI. (**B**) Interaction estimates between combined genotypes with the following adjuvant treatments: Chemotherapy, Tamoxifen, and Aromatase Inhibitor, on BCFI as endpoint. Model adjusts for age at inclusion, BMI, tumour characteristics and adjuvant treatments. (**C**) Hazard ratios of the full multivariable Cox proportional hazard regression for combined *PRLR* genotypes, including an interaction term for radiotherapy on BCFI. The Cox proportional hazard regression was applied to the full multivariable model adjusted for age at inclusion, tumour characteristics, adjuvant treatments and BMI. Ref = reference; HR = Hazard ratio; CI = confidence interval; Pnom = nominal P-value; Padj = Bonferroni-corrected P-value. *P ≤ 0.05, **P ≤ 0.01.,

Supplementary Table S6. (**A**) Interaction estimates between haplotypes and Radiotherapy, on BCFI as endpoint. Model adjusts for age at inclusion, BMI, tumour characteristics and adjuvant treatments. (**B**) Interaction estimates between cp,bined genotypes and Radiotherapy, on death due to any cause as endpoint. Model adjusts for age at inclusion, BMI, tumour characteristics and adjuvant treatments (**C**) Hazard ratios of the competing risk analysis with any new breast cancer event as the primary event and death due to any cause as the competing risk. The Cox proportional hazard regression was applied to the full multivariable model adjusted for age at inclusion, tumour characteristics, adjuvant treatments and BMI. (**D**) Sensitivity Analysis presenting interaction estimates between individual *PRLR* SNPs (top) or combined genotypes (bottom) with radiotherapy on BCFI, and including in the adjusted cox model either breast feeding duration for the first child or total breastfeeding duration. Ref = reference; HR = Hazard ratio; CI = confidence interval; Pnom = nominal P-value; Padj = Bonferroni-corrected P-value. *P ≤ 0.05, **P ≤ 0.01..

Supplementary Table S7. LDlinkr results. Table presents SNPs linked to each of the 5 *PRLR* SNPs. Minor alleles of query SNPs are effect alleles impacting tissue expression of PRLR protein according to eQTL results and correspond to *PRLR* SNPs minor alleles.

Supplementary Table S8. (**A**) Clinicopathological information for all 1980 METABRIC patients included according to *PRLR* expression tertile. (**B**) Full results and overlap analysis of gene ontology results. Description column shows the pathways enriched in either *PRLR*-high tumours (values with positive Normalized Enrichment Score (NES)) or *PRLR*-low tumours (negative NES values).

Supplementary Table S9. Conditional analysis results. TFs presented were seen to be significantly modulated by PRLR, either negatively (Mode = -1) or positively (Mode = 1).

Supplementary Table S10. Master regulators of subgroups of patients with/without radiotherapy and further stratified according to PRLR tertiles and prognosis (BCFI > or ≤ 5 years). Information of conditional modulation by PRLR and regulon status (active or repressed according to GSEA-2T) are also presented.

Supplementary Figure S1

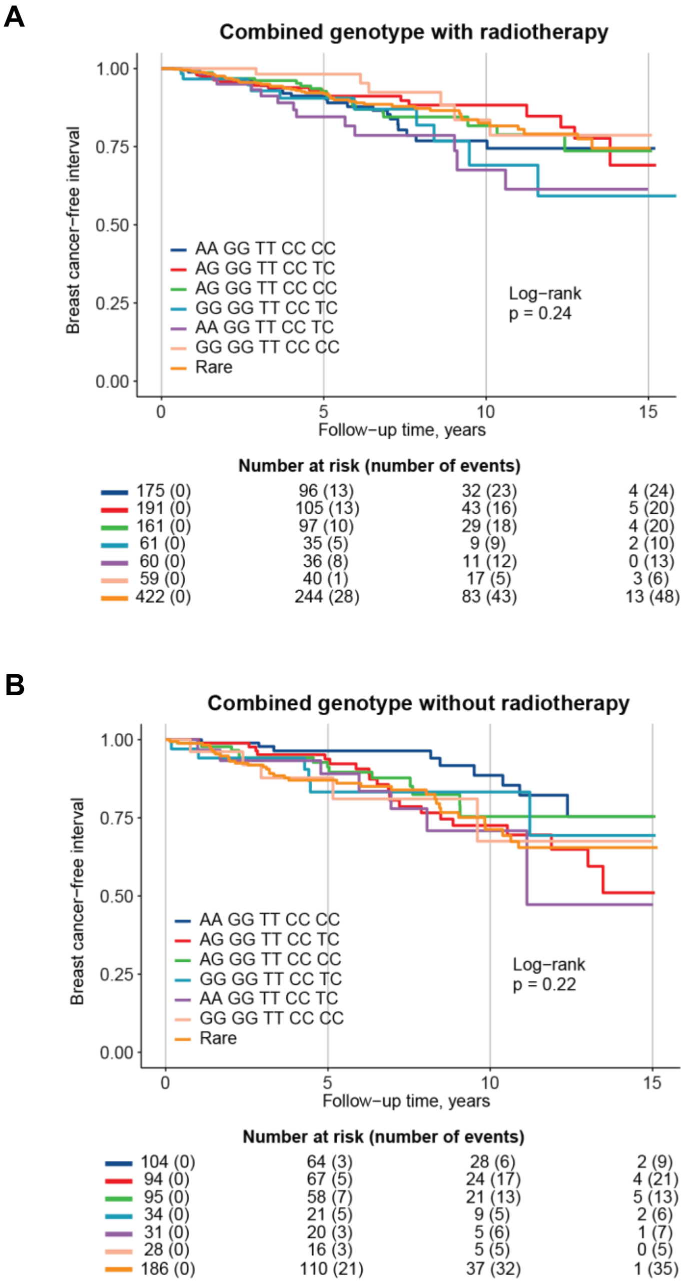

Supplementary Figure S2

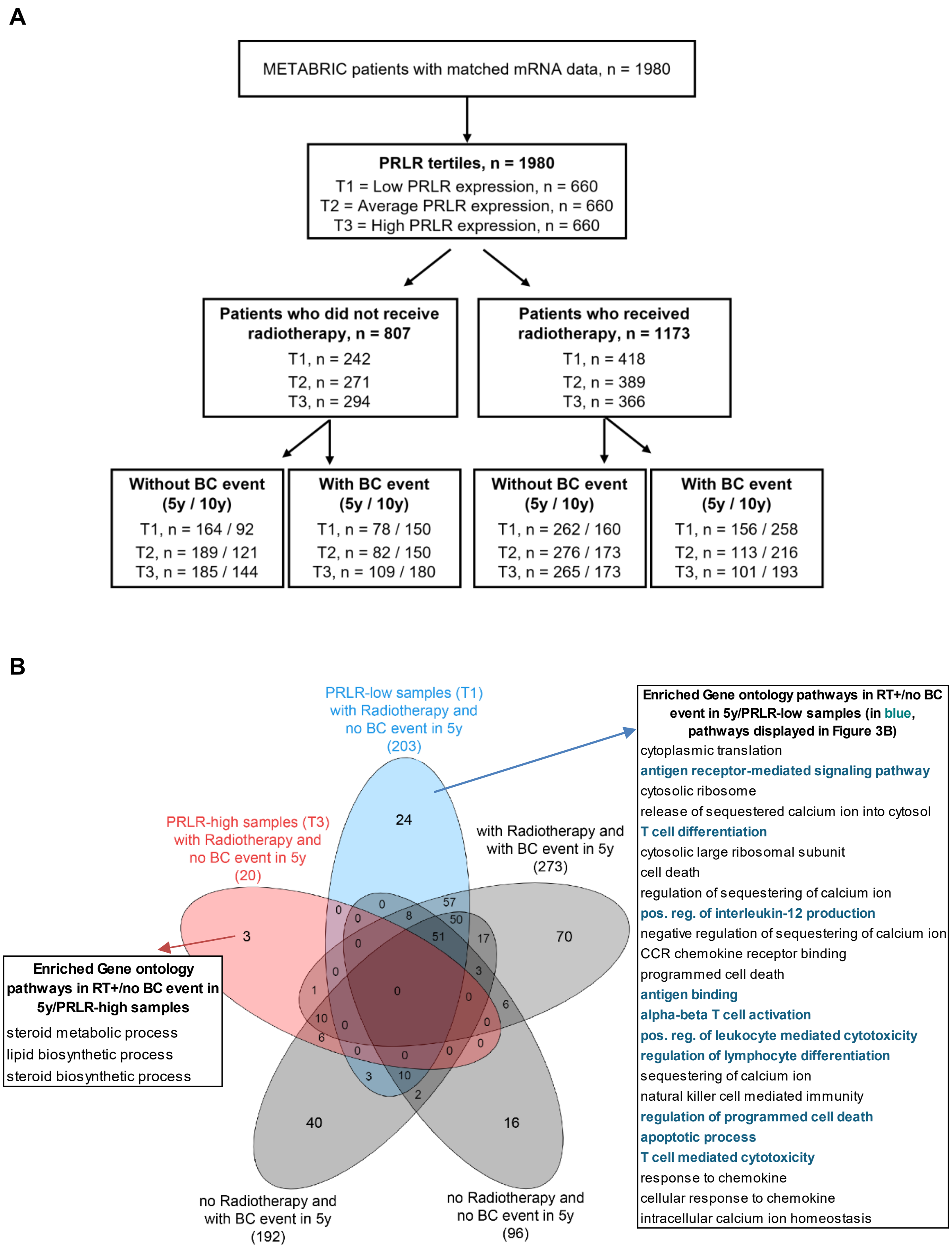

Supplement: Supplementary file 1 [file cancers-17-02378-s001.zip › Supplementary_Figures and_Legends.pdf]
